# The Emergence of Novel Versus Known Three-Dimensional Structures from Random Sequences

**DOI:** 10.64898/2025.12.04.691947

**Published:** 2025-12-07

**Authors:** Rose Yang, Hyunjun Yang, Anton Davydenko, Zack Mawaldi, Rian Kormos, Dru Myerscough, Yibing Wu, William F. DeGrado

## Abstract

It has been hypothesized that while random sequences are unlikely to fold into proteins of the length of globular proteins, repeated random sequences are more likely to adopt stably folded structures, with implications for molecular evolution. We used structure prediction methods to determine the foldability of approximately 120-residue sequences composed of 5-to 60-residue random repeats. With repeats of less than 30-residues, sequences were frequently discovered (1–12%) that fold with high confidence. For less than 60-residue repeats, we frequently observe β-solenoids, similar to those seen in natural proteins. We observe solenoids stabilized by apolar packing as well as ones stabilized by polar interactions with Ca ^2+^ in the core of the structure as in natural RTX domains. Helical bundles were observed with high frequency when insertions or deletions (INDELs) were included between blocks of repeating sequences. We also observed a new super-secondary structure consisting of a tightly wound α-helical screw, and experimentally confirmed its stability and structure by CD spectroscopy and X-ray crystallography. Thus, structure predictors can discover structures that are well out of the distribution of the data upon which they were trained. Beyond 40-residue repeat lengths very few sequences were predicted to fold. The small number of structures we observed were representative of well-established major classes of tertiary structures; greater sampling would be needed to discover novel structures from a random distribution. These studies illuminate dark matter regions of protein structure space and support previous predictions that proteins evolved through the assortment of shorter peptide sequences.

**Significance statement:**

The availability of powerful and accurate programs for predicting protein three-dimensional structures enables one to ask fundamental questions concerning the origin of folded functional proteins during evolution. We show that 120-residue proteins composed of random sequences repeated in tandem are predicted to be much more likely to fold than fully random proteins. These studies validate previous predictions that proteins evolved through the repetition and assortment of short peptide sequences. Also, some of the predicted structures represent novel conformations, which were confirmed experimentally. These findings advance our understanding of molecular evolution and have implications for design of novel proteins.

## Full Text Availability

The license terms selected by the author(s) for this preprint version do not permit archiving in PMC. The full text is available from the preprint server.

